# Correlates of COVID-19 vaccination intentions and opinions about mandates among four groups of adults in South Africa with distinct vaccine intentions: evidence from a large national survey

**DOI:** 10.1186/s12889-023-16584-w

**Published:** 2023-09-11

**Authors:** Katherine Eyal, Lindokuhle Njozela, Timothy Köhler, Kim Ingle, Timothy Brophy, Alison Buttenheim, Brendan Maughan-Brown

**Affiliations:** 1 Southern Africa Labour and Development Research Unit, School of Economics, University of Cape Town, Cape Town, South Africa; 2 Development Policy Research Unit, University of Cape Town, Cape Town, South Africa; 3 Department of Family and Community Health, University of Pennsylvania School of Nursing, 416 Fagin Hall, 418 Curie Blvd, Philadelphia, PA 19104 USA

**Keywords:** Vaccine intentions, Vaccine hesitancy, COVID-19, Trust, Attitudes, Mandates

## Abstract

**Introduction:**

Despite a high number of recorded COVID-19 infections and deaths in South Africa, COVID-19 vaccine coverage remained low in March 2022, ten months into the national vaccine roll-out. This study provides evidence on the correlates of vaccine intentions, attitudes towards vaccination and opinions about mandates.

**Methods:**

We used data from the second COVID-19 Vaccine Survey (CVACS), a telephone survey conducted February-March 2022 among 3,608 South African adults who self-reported not being vaccinated against COVID-19. The survey instrument was designed in consultation with government, policymakers, and civil society; and segmented the sample into four distinct groups with different vaccine intentions (synonymous with vaccine hesitancy levels). Kruskal-Wallis and Mann-Whitney tests were used to examine the sociodemographic characteristics, attitudes and behaviours associated with the different vaccination intentions groups. Thematic coding of responses to open-ended questions elicited insights on reasons for not being vaccinated and attitudes towards mandates.

**Results:**

Intentions to get vaccinated were greater among individuals with lower socio-economic status (Mann–Whitney Z = -11.3, *p* < 0.001); those believing the vaccine protects against death (Kruskal–Wallis Χ^2^ = 494, *p* < 0.001); and those who perceived themselves at risk of COVID-19-related illness (Χ^2^ = 126, *p* < 0.01). Vaccine intentions were lower among individuals who believed that the vaccine causes death (Χ^2^ = 163, *p* < 0.001); believed that the vaccine is unsafe for the babies of pregnant/breastfeeding mothers, or the chronically ill (Χ^2^ = 123, *p* < 0.01); those not trusting government health information about COVID-19 and the COVID-19 vaccine (Kendall’s τ = -0.41, *p* < 0.01); and those in opposition to mandates (τ = 0.35, *p* < 0.001). Only 25% supported mandates, despite 48% thinking mandates would work well, with 54% citing individual rights as their main reason for mandate opposition.

**Conclusion:**

The profile of individuals not vaccinated against COVID-19 as of March 2022 varied markedly by self-reported vaccination intentions, underscoring the importance of tailored demand-creation efforts. This paper highlights several factors which differ significantly across these groups. These findings could inform the design of future vaccination campaigns, potentially increasing their likelihood of success. This is an important policy objective given widespread vaccine hesitancy, and further work is required on this topic. Mandates remain an option to increase coverage but need to be carefully considered given extensive opposition.

## Introduction

By August 2022, 6.4 million people were estimated to have died from COVID-19 globally, with 584 million infections recorded [1]. Despite the proven efficacy of vaccines in reducing transmission and COVID-19 deaths [2–7], vaccine hesitancy was widespread in many countries [4, 7–14]. Insufficient demand resulted in the disposal of numerous expired vaccine doses globally [15, 16].

South Africa had experienced 5 waves of COVID-19 infection, with 102,000 officially recorded deaths and 4 million recorded infections by early August 2022 [1], with actual cases estimated to be substantially higher [17]. South Africa faced a persistent concern of high rates of chronic illnesses [18], identified as COVID-19 comorbidities [11, 16, 17, 19]. Following a clinical trial that commenced on the 17th February 2021 [20], in which 479,768 health workers were vaccinated, a phased national vaccine rollout began on the 17th of May 2021. Individuals aged 60 and above were initially eligible, followed by those in age categories 50-59, 35 to 49, 18 to 34, and then 12 to 17.

Prior to the COVID-19 pandemic, hesitancy to vaccines in general was documented globally [8, 12, 21, 22], and identified as a key threat to global population protection [13, 23]. The determinants and degree of vaccine hesitancy (defined as delayed acceptance or refusal to take up available vaccines) have differed significantly both within and across countries [7, 8, 18, 24–28]. In addition, vaccine hesitancy has varied by disease, with higher acceptance for more established vaccines (Measles-Mumps-Rubella, Diphtheria-Tetanus-Pertussis), and more hesitancy observed for others, particularly for newer vaccines (human papillomavirus (HPV), meningococcal, pneumococcal, influenza) [29–31]. Low vaccine take-up has been associated with institutional mistrust [7, 13, 32, 33], conspiracy and misinformation [7, 33–35], fear of side effects [7, 21, 27, 35–37], lack of access and knowledge gaps [32, 35], lack of belief in efficacy [7, 12, 24, 38], resistance in communities, and low perceived risk of COVID-19 infection [7–9, 13, 16, 20–22, 25]. Hesitancy has also differed with age, gender, and other demographic factors [5, 7, 12, 28, 39].

For COVID-19 vaccines specifically, many of the factors above have amplified due to the speed of vaccine development [3–5, 19], which have resulted in perceptions that the vaccines are insufficiently tested, unsafe, and ineffectual [7–9, 11, 12, 14, 18, 27, 35, 37, 40]. Despite high initial rates of COVID-19 vaccine acceptance in early to mid 2021 [9, 21, 24, 28], hesitancy increased as milder variants of COVID-19 [4, 16, 41–43] have been more easily transmitted, even among the vaccinated [2, 3, 19, 40, 42, 44, 45]. Perceptions of low vaccine efficacy have persisted, despite the continued effectiveness of vaccines against serious illness [4, 21, 42]. The need for boosters [3, 4, 6] has also complicated vaccine demand creation efforts.

COVID-19 vaccination rates in Africa have remained disturbingly low, and far lower than in high-income countries [6, 16]. At the time of CVACS Surveys 1 and 2, quantitative evidence on vaccine hesitancy on the continent was limited [32], with no nationally representative studies having taken place [17, 27, 34]. More recently, additional surveys by the World Bank, the African CDC, UNICEF, and other bodies, in multiple African countries have built on this knowledge base [46–48], including similar rapid surveys in Eastern and Southern Africa [49]. Globally, deeper psychographic research [13, 20, 23] along a continuum of vaccine hesitancy [23, 25], is required to better inform demand creation strategies [21, 23]. Incentives, for example, have been shown to increase influenza [33] and COVID-19 vaccination rates [50]. However, these may be less effective among the more hesitant [51], and may backfire and increase hesitancy if introduced without sufficient knowledge of local context [23, 45].

Despite early indications that South African vaccine acceptance would be high [8, 12, 18, 34], by early December 2021 when the Omicron variant was announced, only 15 million South Africans had received at least one dose of the vaccine. This constituted a vaccination rate of only 25% among the adult population [52]. Initially poor access restricted vaccine uptake [51]. However, despite increased vaccine access and extensive national demand creation activities [21, 23], vaccine hesitancy persisted [37].

Our study adds to the limited evidence on COVID-19 vaccine hesitancy in South Africa [12, 18, 34, 53, 54]. Data from the COVID-19 Vaccine Survey (CVACS) – a national policy responsive survey of vaccination intentions in the Omicron period in a large and diverse sample – is used to assess the correlates of COVID-19 vaccine hesitancy, and how these vary across groups with different vaccination intentions. Quantitative and qualitative findings from CVACS Survey 2 (February–March 2022) improve our understanding on vaccination intentions, vaccine efficacy and safety beliefs, trust in government health information and attitudes to mandates.

## Methods

### Data source

CVACS [55, 56] was conducted as a policy responsive telephone survey, using a multi-pronged approach to questionnaire design. The survey instrument was informed following in-depth engagement with policy makers and stakeholders in the vaccine demand creation space, as well as a crowd-sourced approach for potential questionnaire items. The survey collected data on demographics, socio-economic and health indicators, reasons behind non-vaccination, attitudes and intentions to vaccinate, and attitudes to vaccine mandates [57]. The survey instrument was translated into all official languages.

#### Study design

 Adults (aged eighteen or older) unvaccinated against COVID-19 (whether fully or partially) were eligible for the survey. Vaccination status was self-reported. The survey took place telephonically, with Survey 1 interviews from 15^th^ November-15^th^ December and Survey 2 interviews from 23^rd^ February-25^th^ March 2022. The sample frame was a large credit bureau database, including individuals who had applied for credit, regardless of the outcome, and individuals who had had a credit check. The sample was primarily stratified across province, population group, geographic area type (metropolitan municipalities, non-metropolitan urban municipalities, non-metropolitan rural municipalities) and income. We were able to obtain access to this database through the GeoTerraImage (GTI) 2021 sampling frame [58], which was linked at the enumeration area level. We chose this sampling frame because of its broad coverage across predicted correlates of vaccination behaviour, and its multiple strata, while recognizing that it shaped the generalizability and representativeness of our study with its slightly higher socio-economic profile (as discussed in the limitations below).

The neighbourhood lifestyle index (NLI) was used to measure income, with NLI groups of 1–2, 3–4 and 5–10. The NLI is based the classification of neighbourhoods by income indicators and various lifestyle characteristics. Area and NLI data were obtained from the GeoTerraImage (GTI) 2021 sampling frame [58], Age categories (defined according to the COVID-19 vaccination age groups: 18–34, 35–49, 50–59, 60 +) and gender were used as further explicit stratification variables. Design weights were calculated to account for sample selection and non-response, but weighted CVACS data is not nationally representative of all unvaccinated individuals in South Africa.

CVACS Survey 2 interviewed 1,722 (still unvaccinated) respondents of the original 3,510 Survey 1 sample, and a top up sample of 2,222 unvaccinated respondents (from the original sampling frame). The final realised sample contained 3,608 unvaccinated respondents. Our analysis is based on CVACS Survey 2 data.

### Ethical considerations

Before commencement of the study, ethics clearance was obtained from the Commerce Faculty ethics committee at the University of Cape Town, South Africa (REF REC 2021/11/007). Prior consent was given by potential participants in the sampling frame to receive calls of this nature. All potential participants were informed as to the nature of the study (a telephone survey on individual opinions on COVID-19 and vaccinations). Subsequently only those who voluntarily agreed to be part of the study were included. Verbal consent was obtained, which was recorded. All calls were recorded with the participant’s permission. All information collected during the CVACS study was kept confidential and anonymous. The study was performed in accordance with the Declaration of Helsinki and all participants gave informed consent. See related files for the full ethics clearance and participant consent preamble from the CVACS questionnaire.

#### Sample characteristics

Data for several participant demographic characteristics was collected, including age, gender, education, mental and physical health, household characteristics (including household size, socio-economic indicators, and location), and COVID-19 related information. Mental health was measured as the presence of self-reported depressive symptoms according to the PHQ-2 score (PHQ-2 > 2) [59]. Chronic illness referred to human immunodeficiency virus (HIV), lung or heart conditions, hypertension, or diabetes.

#### COVID-19 vaccination intentions

To assess different vaccination intentions, the following question was asked of respondents: *“Regarding the COVID-19 vaccine, do you plan to: 1. get it as soon as possible, 2. wait and see, 3. only if required (for example, if it is required for school or work) or 4. definitely not get it?”*. The “don’t know” (90 respondents, less than 3% of the sample) and “refused” (only 4 respondents) were excluded from analyses.

### Statistical analysis

Analysis was performed using Stata SE V.17. Design-weighted estimates are reported. Significance was set at *p* < 0.05, with significance levels reported as follows: * for *p* < 0.01, ** for *p* < 0.01 and *** for *p* < 0.001. Based on the patterns observed in many of the reported beliefs, we treat the self-selected intentions categories as an ordinal measure in the statistical analysis. Results were substantively the same in sensitivity analysis that treated the intentions groups as categorical.

#### COVID-19 vaccination intentions and associated factors

The Kruskal–Wallis test (Chi-squared values reported) was used to test relationships between vaccination intentions and a set of beliefs about the likelihood of getting vaccinated in the near future, vaccine efficacy, and vaccine safety. These categorical variables had answers of “Yes”, “No” and “Don’t know”, with a non-trivial proportion of “Don’t know” responses (who were thus included in the analysis). Respondents who refused to answer these questions (fewer than 0.05% of respondents) were excluded. The Mann–Whitney Rank sum test (Z-scores reported) was used to test for significant differences by intention group for 2 binary indicators of socio-economic status and chronic illness. Respondents with “Don’t know” and “Refused” answers for these 2 indicators were excluded from the analysis.

#### Reasons for not getting vaccinated

Participants were asked: “*I will now ask about some potential reasons why you are not yet vaccinated. This may or may not include the reason you already mentioned. Please answer yes or no to each of the following.*”. Multiple reasons could be answered in the affirmative. These were coded into binary indicators, which were tested for significant association with vaccination intentions using a Mann–Whitney Rank sum test. Z-scores and significance levels are reported. Refusals (fewer than 20 respondents, 0.5% of the sample) were excluded, while answers of “Don’t know” were included.

#### Main reason for not vaccinating: qualitative responses

Respondents were asked the open-ended question: “*What is the single biggest reason that you are not yet vaccinated?*”. Answers were coded into salient themes or categories, which were informed by the question responses themselves, the literature, and applicable behavioural theory. To accomplish this, a team of 3 researchers independently analysed 200 responses, and the results were harmonised into one codebook. Following common practice [60], the full set of 3,608 responses was then double coded using the codebook, with differences reconciled using a separate coder blinded to the contradictory codes. Remaining mismatches at this point were coded as uncategorised (approximately 6% of responses). Thematic analysis of the final set of codes was performed.

#### Mistrust and mandates

We test for significant association between vaccination intentions and measures of trust of government COVID-19 health information, and attitudes to mandates. These ordinal measures had a minimum of “Don’t know” answers. Fewer than 0.5% of respondents refused to answer these questions. We report the Kendall rank correlation coefficient, τ, and significance levels.

#### Main reason for strongly opposing a mandate: qualitative responses

Following the same method of thematic coding used above for main reason for being unvaccinated, we analysed responses to an open-ended question about mandate attitudes: “*Please can you tell us the main reason why you feel that way about vaccine requirements or mandates?*”. All respondents were asked this question. We report the main themes ranked in order, for the sample of respondents who strongly opposed a mandate.

## Results

### Sample characteristics

Consistent with other telephone surveys, our sample displayed above average socio-economic status relative to the South African population, but was still characterised by considerable financial hardship and poor health (Table 1).

**Table 1:** Summary statistics: individual and household characteristics

**Sample Characteristics**	**n**	**Mean/%**
**Vaccine rollout age categories**		
Aged 18 to 34 (%)	3,558	54.7
Aged 35 to 49 (%)	3,558	28.8
Aged 50 to 59 (%)	3,558	9.2
Aged 60 or above (%)	3,558	7.3
**Demographic characteristics**		
Age (years)	3,557	37.5
Female (%)	3,607	46.9
Has a matric certificate (%)	3,545	67.7
Has a tertiary qualification (%)	3,096	49.4
Earned money recently (%)	3,608	60.0
Has medical insurance (%)	3,556	24.0
Has a chronic condition (%)	3,541	21.5
Depressive symptoms present (PHQ > 2) (%)	3,555	30.1
**Religion is important to me:**		
Not at all (%)	3,513	6.8
Unimportant (%)	3,513	4.3
Yes (%)	3,513	29.2
Very important (%)	3,513	59.7
**Household characteristics**		
Household size	3,589	4.4
Hunger in the household (past week) (%)	3,588	19.1
Household receives a government grant (%)	3,477	57.3
Household income last month (R)	2,505	11,496
Household income > = R5000 (%)	3,082	47.4
Own a running vehicle (%)	3,578	46.2
**Household lives in:**		
Traditional area (%)	3,573	14.1
Township/informal (%)	3,573	46.8
Formal residential (%)	3,573	30.3
Farm/smallholding (%)	3,573	8.8
**COVID-19**		
I have had COVID-19 (%)	3,496	18.6
Lives with vaccinated person (%)	3,590	45.8

Weighted descriptive statistics of individual and household characteristics are reported, as mean values where appropriate (age, household size, and household income), or percentages. The presence of depressive symptoms is defined as a PHQ-2 score above 2. Chronic illnesses refer to any of HIV, lung condition, heart condition, high blood pressure, or diabetes. Examples given for government grants included the state old age pension, child support grant, and the COVID-19 social relief of distress grant. CVACS Survey 2. Authors’ own calculations

The sample had above-average education levels (68% had matriculated, 49% had a tertiary qualification). Although respondents reported higher household incomes than many South Africans, with 47% of respondents living in households with monthly income above R5,000 (approximately 300 United States (US) dollars), hunger was prevalent in 19% of the respondents’ households and 57% reported their household received government welfare grants. 22% of respondents reported having a chronic condition and 30% presented with depressive symptoms.

The age and gender distributions of respondents was 55% (18-34), 29% (35-49), 9% (50-59) and 7% (60-plus); 47% of respondents identified as female. 47% of respondents lived in township or informal areas. Approximately 19% of the sample reported having had COVID-19, and 46% lived with a vaccinated person. The vast majority held religion as important or very important to them (89%).

### COVID-19 vaccination intentions and associated factors

Study participants were mostly reluctant to vaccinate. Only 19% intended to vaccinate as soon as possible, 20% intended to wait and see, 25% only if required, and the majority, 37%, reported they definitely would not (Table 2). 56% did not think they would be vaccinated by May, with this figure at 86% for the “definitely not” group. (Kruskal–Wallis Χ^2^ = 661, *p* < 0.001). 20% of the “as soon as possible” group thought they would get very sick with COVID-19 in the next year, compared to only 3% of the “definitely not” group (Χ^2^ = 126, *p* < 0.001).

**Table 2:** Differences in vaccination beliefs and attitudes across vaccination intention groups

		**Do you intend to get vaccinated?**				**Z or χ**^**2**^	**Sig**
**Intentions group:**	**All**	**As Soon as Possible**	**Wait and See**	**If Required**	**Definitely Not**		
**Percentage of sample**	100	18.8	19.7	24.6	36.9		
**Number of observations**	3608	680	712	828	1294		
**Will you be vaccinated by May?**						χ^2^ = 661	^***^
Yes (%)	29.0	77.4	31.6	25.3	4.5		
No (%)	55.8	11.5	40.8	58.5	85.7		
I don’t know (%)	14.5	9.8	26.7	15.7	9.1		
**I will get very sick with COVID-19 this year:**						χ^2^ = 126	^***^
Yes (%)	8.0	20.2	7.4	6.6	3.3		
No (%)	74.2	54.7	70.9	76.6	85.4		
I don’t know (%)	17.8	25.1	21.6	16.8	11.2		
**Vaccine Efficacy and Safety**							
**The vaccine will stop me dying from COVID-19:**						χ^2^ = 494	^***^
Yes (%)	31.5	72.0	36.7	28.5	11.2		
No (%)	61.0	22.7	52.2	64.2	83.7		
I don’t know (%)	7.5	5.3	11.1	7.3	5.2		
**I believe the vaccine can kill you:**						χ^2^ = 163	^***^
Yes (%)	28.1	12.2	19.4	29.1	41.3		
It might (%)	29.7	25.2	36.1	32.6	26.2		
No (%)	33.5	57.3	36.2	31.6	21.8		
I don’t know (%)	8.7	5.3	8.3	6.8	10.7		
**The vaccine will harm or keep people healthy:**						χ^2^ = 171	^***^
Healthy (%)	30.6	70.6	33.4	25.0	12.9		
Neither (%)	12.7	8.0	16.9	13.9	12.2		
It will harm (%)	40.4	12.7	30.9	41.8	60.3		
I don’t know (%)	16.4	8.6	18.9	19.4	14.6		
**The vaccine is safe for the babies of pregnant/breastfeeding mothers:**						χ^2^ = 123	^***^
Yes (%)	21.8	43.8	25.8	17.7	10.7		
No (%)	56.1	33.0	49.5	59.6	70.6		
I don’t know (%)	22.1	23.2	24.8	22.7	18.7		
**The vaccine is safe for chronic illness:**						χ^2^ = 92	^***^
Yes (%)	25.2	51.6	25.2	24.8	11.3		
No (%)	52.8	28.2	48.2	57.1	66.0		
I don’t know (%)	22.1	20.2	26.6	18.1	22.7		
**Socio-economic status & chronic illness**							
Household income > = R5000 (%)	47.4	35.9	40.8	51.3	55.8	Z = -11.3	^***^
Has a chronic condition (%)	21.5	22.3	26.2	21.3	19.0	Z = 2.0	^*^
**Percent responding yes to each potential reason why the respondent is unvaccinated**							
God or the ancestors will protect me (%)	51.7	39.6	52.1	54.6	54.9	Z = -5.5	^***^
Vaccination site is too far away (%)	15.8	31.7	19.0	15.5	6.7	Z = 15.0	^***^
My body is strong enough to fight the disease (%)	64.6	49.0	62.3	68.2	72.9	Z = -13.5	^***^
My religious leader is against the vaccine (%)	8.7	7.9	8.7	7.7	9.9	Z = -1.6	
No time to go get vaccinated (%)	22.7	46.4	26.9	22.7	8.0	Z = 18.6	^***^
My risk of being infected is very low (%)	48.7	36.3	46.7	52.3	54.7	Z = -5.5	^***^

Weighted descriptive statistics of beliefs relating to reasons for not vaccinating, vaccine efficacy, and safety in the full sample, and 4 vaccination intentions groups. These are respondents who intend to get vaccinated: as soon as possible, to wait and see, to get vaccinated only if required to do so, or to definitely not get vaccinated. Test statistics are Kruskal–Wallis Chi-squared or Mann–Whitney Rank Sum Z-scores

Significance levels are reported as follows:^*^ represents 0.01 <  = *p* < 0.05, ^**^ represents 0.001 <  = *p* < 0.01, ^***^ represents *p* < 0.001. Authors’ own calculations. CVACS Survey 2

Beliefs about infection risk, and vaccine efficacy and safety showed stark and consistently significant divergence by vaccination intentions, especially between the “as soon as possible” and “definitely not” groups. A majority (72%) of the “as soon as possible” group believed the vaccine prevented death from COVID-19, compared to only 11% of the “definitely not”s (Χ^2^ = 494, *p* < 0.001). Additionally, 41% of the “definitely not” group believed the vaccine could kill, compared to 12% of the “as soon as possible”s, a significant difference (Χ^2^ = 163, *p* < 0.001). Many more of the “definitely not” group (60%) thought the vaccine would harm people, compared to 13% of the “as soon as possible” group (Χ^2^ = 171, *p* < 0.001). Vaccination intentions were significantly associated with the belief that the vaccine is safe for the babies of pregnant or breastfeeding mothers (Χ^2^ = 123, *p* < 0.001), or safe for those with a chronic illness (Χ^2^ = 92, *p* < 0.001), but only a minority reported holding these beliefs (22% and 25% respectively). The majority perceived their risk of becoming very ill from COVID-19 as low, while a minority believed the vaccine is safe or effective. Little difference existed across the groups in terms of rates of chronic illness, with 21.5% in the “as soon as possible” group reporting suffering from a chronic illness, with a comparable 19% in the “definitely not” group.

Vaccination intentions varied significantly by both socio-economic status and physical health. Only 36% of the “as soon as possible” group reported household income above R5000, compared to 56% of the “definitely not” group (Z = -11.3, *p* < 0.001). The prevalence of chronic illness similarly differed by vaccination intentions, although in a more limited range: from 19% for the “definitely not” group, to 26% of those intending to “wait and see” (Z = 2.0, 0.01 <  = *p* < 0.05).

### Reasons for not getting vaccinated

When asked why they were unvaccinated, and given the opportunity to choose multiple reasons, the top three reasons chosen by all respondents were a “body strong enough to fight the disease” (65%), protection from “God or the ancestors” (52%) and a “low risk of being infected” (49%). The opinions of religious leaders were not significantly related to vaccination intentions. Large and significant variation in all other reasons by vaccination intentions was observed, with access concerns highest among the “as soon as possible” group (but low in the full sample), and belief in low risk of COVID-19 infection and a strong immune system most prevalent among the “definitely not”s.

Belief in the protection of God or the ancestors as a reason for not vaccinating was significantly larger for the “definitely not” group at 55%, compared to the “as soon as possible” group (40%) (55%) (Z = -5.5, *p* < 0.001). Two access issues, related to distance or a lack of time, were cited more frequently by the “as soon as possible” group (32% and 46% respectively) compared to the “definitely not”s (7% and 8% respectively, Z = 15.0 and Z = 18.6, *p* < 0.001). Overall, a minority of all respondents reported distance or time as a barrier (16% and 23% respectively).

Belief in a strong immune system (“my body is strong enough”) was highest among the “definitely not” group (73%), but was also reported by 49% of the “as soon as possible” group. This belief varied significantly across intentions (Z = -13.5, *p* < 0.001). Nearly 49% of the sample estimated their risk of being infected with COVID-19 at very low, and this measure varied significantly by vaccination intentions (Z = -5.5, *p* < 0.001).

### Main reason for not vaccinating: qualitative responses

**Fig. 1:**
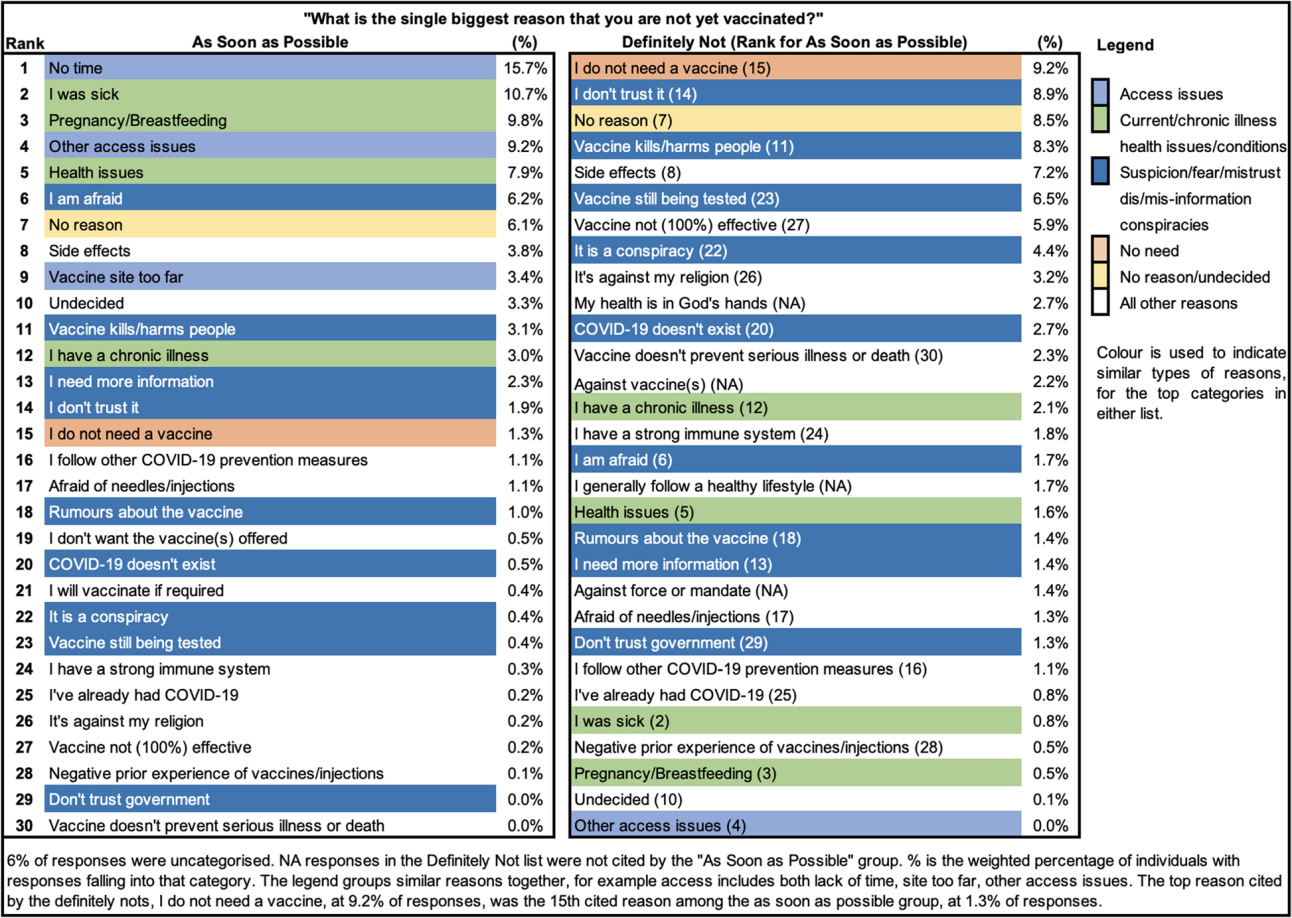
Single biggest reason for not being vaccinated yet in the "As soon as possible" and "Definitely not" vaccination intentions groups

The qualitative evidence showed diametrically opposed views on vaccination across the vaccination intention groups, with very little overlap (Fig. 1). The top 5 most frequently cited reasons by the “as soon as possible” group (totaling to more than 50% of responses) were concentrated in access and health related issues, which were scarcely mentioned by the “definitely not”s. Not needing or not trusting a vaccine was cited nearly 8 times as much by the “definitely not” group as the “as soon as possible”s. Conspiracies and fear were more common among the “definitely not’ group, but these were also cited by the “as soon as possible” group too, although in much lower proportions.

For the “as soon as possible” group, logistical and access reasons were predominant (16% cite no time, 11% cite being sick, 9% and 8% cite general site access and health issues respectively, and a further 3% state the vaccine site was too far). In addition, reflecting incorrect beliefs about vaccine safety, reasons for non-vaccination included being pregnant or breastfeeding (10%) or having a chronic illness (3%).

For the “definitely not” group, the top 4 themes differed in frequency by less than 1 percentage point and reflected dissimilar motivations. The top reason, “I do not need a vaccine" (9%) and “No reason” (9%) indicate certainty in the decision not to vaccinate. “I don’t trust it” (9%), “Vaccine kills or harms people” (8%) or “Side effects” (7%), “Vaccine still being tested” (7%) or “Vaccine not 100% effective” (6%) or “It is a conspiracy” (4%), reflected a major portion of this group who were distrusting, afraid, or skeptical. A deep divergence in views was apparent across intentions groups—the most frequent theme (“No time”) cited by 16% of the “as soon as possible” group did not feature in the 30 most frequently observed themes among the “definitely not” group. Similarly, the most frequently reason cited by the “definitely not” group (“I do not need a vaccine”, 9%) was only the 15th reason (1%) for the “as soon as possible” group. Similar differences can be seen for beliefs in vaccine efficacy, fear of harm from the vaccine, and other themes. 4 themes cited by the “definitely not” group were not mentioned at all by the “as soon as possible” group (“NA”).

### Mistrust and mandates

Trust in government information on COVID-19 was low, and this lack of trust differed significantly by vaccine intentions (Table 3). 40% did not trust information on COVID-19 from the government at all, with the level of mistrust largely and significantly varied across the intentions groups, ranging from 10% in the “as soon as possible” to 64% in the “definitely not” group (Kendall’s τ = -0.41, *p* < 0.001). Support (including strong support) for mandates was low (25%), and varied significantly, ranging from 59% for “as soon as possible” to 9% for “definitely not” (τ = 0.35, *p* < 0.001), despite that 48% thought mandates would work fairly well or very well. These beliefs in the potential effectiveness of mandates differed significantly by intentions group (τ = 0.28, *p* < 0.001), and was lowest (29%) among the “definitely not” group.

**Table 3:** Differences in trust and attitudes to mandates across vaccination intentions groups

		**Do you intend to get vaccinated?**				**Kendall’s Tau**	**Sig**
**Intentions group:**	**All**	**As Soon as Possible**	**Wait and See**	**If Required**	**Definitely Not**		
**How much do you trust information on COVID-19 from the government?**						τ = -0.41	^***^
A lot (%)	22.9	54.8	25.2	18.4	8.9		
A little (%)	34.4	34.0	44.0	40.6	24.5		
Not at all (%)	40.0	9.5	28.6	37.5	63.8		
I don’t know (%)	2.7	1.7	2.1	3.4	2.8		
**How much do you support a mandate?**						τ = = 0.35	^***^
Strongly support (%)	11.2	32.5	10.6	6.6	4.1		
Support (%)	13.6	26.9	16.0	14.9	4.8		
Oppose (%)	23.2	18.1	26.6	23.8	23.9		
Strongly oppose (%)	49.9	21.0	45.4	52.1	65.9		
I don’t know (%)	2.1	1.5	1.4	2.5	1.3		
**How well will a mandate work?**						τ = = 0.28	^***^
Very well (%)	25.4	48.9	29.4	25.6	11.7		
Fairly well (%)	22.3	23.6	25.1	25.8	17.7		
Not at all well (%)	46.3	21.3	40.3	43.3	65.2		
I don’t know (%)	6.0	6.2	5.3	5.4	5.3		

Weighted descriptive statistics of beliefs relating to trust in government COVID-19 information, and mandates in the full sample, and 4 vaccination intentions groups. These are respondents who intend to get vaccinated: as soon as possible, to wait and see, to get vaccinated only if required to do so, or to definitely not get vaccinated. Tests of association between vaccination intentions and these beliefs are reported from Kendall’s Tau tests

Significance levels are reported as follows: ^*^ represents 0.01 <  = *p* < 0.05, ^**^ represents 0.001 <  = *p* < 0.01, ^***^ represents *p* < 0.001. Authors’ own calculations. CVACS Survey 2

### Main reason for strongly opposing a mandate: qualitative responses

Seven hundred eighty-eight and 1,665 respondents opposed or strongly opposed mandates. More than half of the 1,665 respondents strongly opposed to mandates gave the reason that they felt it was the individual’s right to decide to be vaccinated. Fears of harm from the vaccine, and feeling that vaccines were not needed or were not effective also emerged as respondent themes, but to a much lesser degree (see Table 4).

**Table 4:** Reasons behind lack of support for mandates

**Why do you feel that way about mandates?**	**%**
It is the individual’s right to decide	54.1
Vaccine(s) are not needed	5.2
Fear of harm from vaccines/heard negative rumours	4.7
Vaccines are not (100%) effective	4.6
President or Government said vaccines wouldn’t be mandated	4.6
Mandates limit freedom	3.3
Vaccines developed too fast/not tested/lack evidence or data	2.2
Do not believe in COVID-19 or the vaccine	1.8
Distrust information on COVID-19 or vaccines	1.6
Too sick/can’t get the vaccine	1.4
Mandates/vaccine are a conspiracy	1.3
Mandates do not consider religious/cultural differences	1.3
Vaccines are unfair if used as a condition of employment	1.2
Distrust in government/political motivation	1.1
People lack (correct) information about vaccines/COVID-19	1.0
Vaccines discriminate against and stigmatise the unvaccinated	1.0
Mandates will not work	0.5
Government should prioritize other things	0.2
**N**	1,665

CVACS Survey 2. Thematically coded main reasons given to open ended question about why the person is strongly opposed to mandates. 6.5% of responses are uncategorised, and a further 1.5% had no reason or did not know. 0.7% of these respondents showed a misunderstanding of the question and gave answers in support of mandates

The remaining reasons given by respondents were scattered over several themes (with no clear concentration in any one category), although many were very similar to the reasons given for not vaccinating among the “definitely not” group in Fig. 1. 5% thought vaccines were not needed, nearly 5% feared harm from vaccines, and nearly 5% thought vaccines were not 100% effective. Referring to a previous speech by President Cyril Ramaphosa, 5% were strongly opposed as the president had said vaccination would not be mandated. Mandates limiting freedom, and vaccines being insufficiently tested were the last meaningfully substantial reasons given (3% and 2% respectively).

## Discussion

### Findings

Using a broad and diverse sample of adults, CVACS provides estimates of vaccination intentions and their correlates in the Omicron era. The results indicate a rapid change in the proportion of vaccine hesitant individuals early in the vaccine rollout. In May 2021 70% of South African adults were willing to get a vaccine [9, 18]. In contrast, by March 2022, only a small proportion of a large and diverse sample of unvaccinated South African adults intended to be vaccinated as soon as possible, despite the reduction in barriers to access [43]. This finding highlights the need for continuous surveillance as rapid changes in the profile of vaccine hesitancy among populations unvaccinated need to be accompanied by rapidly adapting demand creations strategies.

At this stage in the rollout, high-income and higher educated study respondents were significantly less likely to have the intention to get vaccinated. These results accord with other smaller South African studies [8, 12, 39], but are in contrast to samples in the US where health literacy and willingness to vaccinate have been positively correlated with income and education [9, 11, 13, 28]. This finding indicates that access barriers may not be the dominant force in vaccine hesitancy, and that access to vaccines is necessary but not sufficient to guarantee vaccination, especially among the wealthier in the CVACS sample.

In March 2022, this large sample of unvaccinated South Africans did not feel they were at high risk of contracting COVID-19 in March 2022, had low belief in the efficacy of the vaccine, and had many concerns about vaccine safety. Incorrect beliefs about whether certain groups were at risk from the vaccine were common. These findings indicate high risk groups in South Africa, such as those with tuberculosis or HIV, could be less likely to be vaccinated, a finding which accords with other South African and African studies [16, 18], but is in contrast to findings from other countries [28]. In general, attitudes to vaccination in the CVACS study are similar to those in other African countries, for less recent as well as newer vaccines such as Ebola and COVID-19 [29]. This study provides particular value by adding to a limited literature of attitudes towards vaccination in Africa [29], which could help to design effective strategies against future waves of COVID-19 or other vaccine preventable diseases. The qualitative work presented here has shown the decision to vaccinate to be emotive and multi-faceted, implying that approaches to increase vaccination must be done with care, reassurance, and a non-judgmental attitude [4, 7].

The results of the survey should be considered along with the study limitations. Social desirability bias is a known feature of health surveys, particularly those which collect intention to vaccinate data [9, 18, 34]. This phenomenon may also have been present in CVACS: data collection took longer than planned as a high percentage (compared to the national average) of potential respondents reported being vaccinated. Additionally, we report respondent intention to be vaccinated, which may differ from action taken [18, 25, 35, 38]. CVACS Survey 2 was designed to report the attitudes and beliefs of adults in South Africa who by March 2022, had chosen to remain unvaccinated. It is important to note that in this paper we do not report if their views differ from the vaccinated population. However, forthcoming work examining the longitudinal predictors of getting vaccinated between CVACS Survey 1 and Survey 2 finds that intentions are a strong predictor of vaccination behaviour [61].

Our sample may differ from the general population of unvaccinated South Africans given their willingness to participate in the survey [9], as well as their above average socio-economic status [62]. The latter characteristic is typical of telephone [18] and online surveys [9, 12, 24, 34, 35], and was expected given the sampling frame [35]. A face-to-face survey would have been the preferred surveying method, but this was impossible to achieve after South Africa’s 4^th^ wave of COVID-19. Although CVACS is not a prevalence survey, its size and broad coverage across income levels, location type, and other vaccination correlates, and the strength and consistent significance of our results indicate the data may represent many common perceptions and beliefs present in the unvaccinated population in South Africa [11, 12, 35]. These are of clear policy relevance but caution must be applied in any inference, given the limitations noted above.

### Policy recommendations

Perceptions of COVID-19 moving to an endemic phase are becoming more common [9, 41, 63]. If accompanied by less vigorous prevention strategies, this may have deleterious effects on global vaccine coverage [41, 63]. Another area of concern is the possibility of adult vaccine skepticism affecting already declining childhood vaccine programs [12, 33], the re-emergence of other vaccine preventable diseases such as monkeypox (declared a national health emergency in the US in early August 2022) [64], and the potential for low acceptance of new and future vaccines for diseases such as HPV, malaria, HIV, and Ebola [7, 22]. CVACS has provided a model of rapid policy responsive data collection during a health emergency. Future waves of COVID-19 or other vaccine preventable diseases could prove expensive to the South African health system, as very few differences in vaccination intentions are present in our sample among those without or without chronic illnesses. Demand creation efforts should be focused on the chronically ill, particularly on alleviating safety concerns among this group. Mandates may work to increase vaccination rates [9, 33, 42] but are likely to be widely opposed by the unvaccinated. Given the low degree of trust in government information about COVID-19 observed in our unvaccinated sample in March 2022, mandates will need to be framed and enacted carefully [18, 40]. Mandates could be more effective in specific settings like workplaces, schools, and healthcare facilities, or for high-risk groups [7, 33], while being less feasible in other contexts [23, 35]. The role of the government may be to support and facilitate non-governmental organisation (NGO) and private institutions’ mandates/requirements, while maintaining government mandates where feasible.

Increasing vaccine confidence and belief in efficacy can be done both through efforts to combat misinformation [9, 11, 19, 20, 22, 23, 33, 37]. In particular, highlighting the risk and consequences of COVID-19 infection could play a key role to increase vaccination [5, 16, 20, 28, 35, 36] especially in vulnerable groups such as pregnant/breastfeeding mothers [4] or those with chronic illnesses [18]. Our results indicate the potential value in carefully framing public education campaigns [11, 24] and clear communication [7, 12, 19, 25, 38]. These findings accord with other literature that remaining barriers to access need to be addressed [6, 13, 19, 21–23, 33, 37, 43]. Community appreciation and mobilisation are associated with increased trust and uptake [16, 22, 33], as well as emphasis on pro-social norms [28]. Simple and inexpensive nudge strategies to improve vaccination could be used [45], but all interventions must be evidence-based and tailored carefully to different groups to succeed [5, 7, 9, 12, 13, 19, 23, 33]. Campaigns done badly may increase vaccine hesitancy, and understanding motivations behind vaccination intentions is crucial [23], something for which the qualitative component of CVACS is valuable.

Vaccination rates (even without mandates) have been shown to increase with trust in the accuracy of government responses against COVID-19 [27], or with general institutional trust [7, 8, 32], a finding similar to the high degree of government mistrust we observed among our respondents definitely not intending to be vaccinated. Government failure to provide basic services has been associated with vaccine hesitancy [20], as has political discontent [12]. South Africa has a high degree of economic inequality, and recent civil instability and protest in July 2021 which left over 300 people dead [65], imply increasing trust in government may be challenging. This is especially true given the dissonance between the well-resourced vaccine program and other considerably neglected public services [12].

Increasing vaccination coverage remains a global public health priority. We have analysed groups of unvaccinated study participants with different intentions to be vaccinated. These groups have starkly differing motives for not having done so. This suggests there is an ongoing need for evidence-based vaccine demand creation policies which are specifically targeted, particularly to high-risk groups such as the chronically ill and pregnant and new mothers. In in the face of widespread opposition to mandates, interventions to instill trust in government health information, and in the safety and efficacy of vaccines are urgently required.

## Data Availability

The datasets generated and/or analysed during the current study are available in the Datafirst repository, https://datafirst.uct.ac.za/dataportal/index.php/catalog/899. All codes necessary to replicate the results presented in the manuscript will be made available on reasonable request to our corresponding author (katherine.eyal@uct.ac.za).
